# Interactive black blood preparation for interventional cardiovascular MRI

**DOI:** 10.1186/1532-429X-16-S1-P32

**Published:** 2014-01-16

**Authors:** Anthony Z Faranesh, Michael Hansen, Toby Rogers, Robert J Lederman

**Affiliations:** 1 Cardiovascular and Pulmonary Branch, National Heart, Lung, and Blood Institute, Bethesda, Maryland, USA

## Background

Recent work demonstrated the feasibility of using real-time MRI to guide right heart cardiac catheterization. In order to make balloon catheters used in the procedure more conspicuous, the balloons were filled with dilute Gd-DTPA, and non-selective saturation pulses were applied along with acquisition of thick slices. The saturation pulses increased the contrast between blood and the Gd-DTPA filled balloons, but suppressed the signal from the background tissue, effectively removing the anatomical context for image-guidance. The goal of this work was to develop an interactive black blood technique, which would preserve the high contrast of the Gd-DTPA balloon and preserve background tissue signal.

## Methods

A flow-sensitive preparation consisting of 90°-180°-90° RF pulses with symmetric gradients around the 180° pulse was implemented in a real-time pulse sequence, so that it could be interactively switched on and off during the scan. The flow-sensitization direction may be placed relative to either physical or slice axes, and was chosen to saturate flowing blood and preserve background signal. During navigation through the vena cava and the right heart chambers, this direction was selected to be in the head-foot direction, and during navigation in the pulmonary vessels it was selected to be in the anterior-posterior and right-left directions. Images were acquired using a real-time single shot SSFP sequence (flip = 45°; TR/TE = 2.8/1.4 ms). Imaging was performed in swine on a 1.5T Siemens Aera scanner. Institutional approval was obtained for all animal experiments.

## Results

A comparison of the non-selective saturation and black blood preparations are shown below, with the balloon in the inferior vena cava (arrow). The corresponding CNR of the balloon (vs blood) and SNR of the anterior myocardium are listed in Table 1. The contrast of the Gd-DTPA filled balloon is high with both preparations. The myocardial signal is completely nulled with the non-selective saturation pulse, while it is easily visible with the black blood preparation.

**Table 1:** CNR and SNR for images shown in Figure 1

Preparation	CNR (blood-balloon)	SNR (myocardium)
Non-selective	53	23

Black blood	56	68

**Figure 1:**
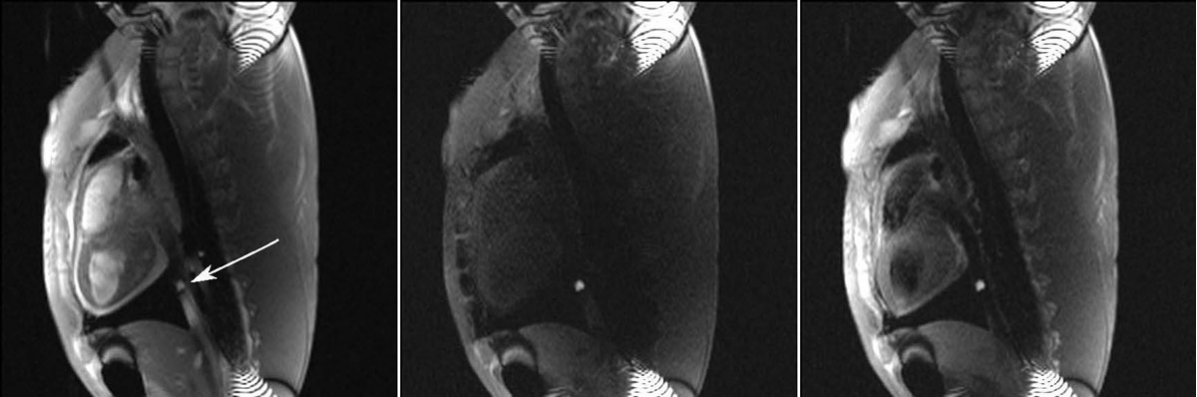
**Images of a Gd-DTPA filled balloon catheter (arrow) in the inferior vena cava in a swine**. (Left) With no saturation, (Middle) non-selective saturation and (Right) black blood saturation. The background signal is preserved with black blood saturation.

## Conclusions

Black blood preparation using flow-sensitizing gradients preserves background tissue signal and imparts high blood-Gd-DTPA contrast. This technique may be useful for for MRI-guided cardiac interventions which use a Gd-DTPA filled balloon for navigation.

## Funding

This work is supported by the NHLBI Division of Intramural Research. Z01-HL006039-01.

